# Responsiveness of Persian 12-Item multiple sclerosis walking scale: a replication study

**DOI:** 10.1186/s13104-023-06316-z

**Published:** 2023-04-04

**Authors:** Soofia Naghdi, Noureddin Nakhostin Ansari, Afarin Haghparast, Amin Nakhostin-Ansari, Maede Khalifeloo, Mahmoud Biglar, Roghie Lotfi, Scott Hasson

**Affiliations:** 1 Sports Medicine Research Center, Neuroscience Institute, Tehran University of Medical Sciences, Tehran, Iran; 2 Department of Physiotherapy, School of Rehabilitation, Tehran University of Medical Sciences, Tehran, Iran; 3 Research Center for War-affected People, Tehran University of Medical Sciences, #594, First floor, Taleghani Ave, Tehran, 14178 Iran; 4 Drug Design and Development Research Center, Tehran University of Medical Sciences, Tehran, Iran; 5 Physiotherapy Clinic, Iran MS Society, Tehran, Iran; 6 Department of Physical Therapy, Augusta University, Augusta, GA USA

**Keywords:** Multiple sclerosis, MSWS-12, Walking, Responsiveness, Minimal clinically important change, Persian

## Abstract

**Objective:**

To re-explore the responsiveness of the Persian version of Multiple Sclerosis Walking Scale-12 (MSWS-12p) to physiotherapy intervention and determine the minimally clinically important change (MCIC). This study followed a prospective cohort design. Patients with MS (PwMS) underwent physiotherapy treatment for 10 sessions. The outcome measures were the MSWS-12p and Timed 25-Foot Walk test (T25-FW). Data was collected before and after ten sessions of physiotherapy. The effect sizes and the area under receiver operating characteristics curve (AUC) and MCIC were calculated.

**Results:**

Thirty PwMS (16 female, mean age 43.07 years) participated in the study. The effect sizes for MSWS-12p were moderate (0.52, 0.64). The change scores of MSWS-12p showed excellent correlation with the dichotomized smallest detectable change (SDC) criterion (Eta coefficient test = 0.84). There was no correlation between the MSWS-12p total change scores and the T25-FW (r = − 0.14, p = 0.45). The AUC was perfect and the MCIC for the MSWS-12p was calculated 10.0 points. The MSWS-12p is responsive and demonstrates changes after physiotherapy. Changes > 10.0 points on MSWS-12p total score should be considered as true improvement after physiotherapy.

## Introduction

Multiple sclerosis (MS) is a prevalent disabling disease of the central nervous system. The prevalence of MS in Iran is 16.5 and 44.8 per 100,000 people in men and women, respectively [1]. Walking dysfunction is a common symptom affecting approximately 80% of patients with MS (PwMS) [2, 3]. Walking dysfunction limits the daily life activities and increases fall risk [3–5]. There are multiple scales for measuring walking in PwMS, including the Timed 25-Foot Walk (T25-FW) [6] and Multiple Sclerosis Walking Scale-12 (MSWS-12) [7].

The MSWS-12 is a self-reported questionnaire that is widely used to measure the impact of MS on walking ability and to detect meaningful changes after treatment of PwMS. Most self-reported questionnaires are in English and for use in other languages must be well translated and adapted culturally for the content validity and equivalency with the source version. The adapted version of a questionnaire needs to be examined for other measurement properties through the process of validation one of which is responsiveness. Validity ensures the scores of a questionnaire are consistent with the construct being measured.

The responsiveness is the ability of a questionnaire to accurately detect any clinical important change, when even small change is occurred. The responsiveness of a questionnaire indicates that the real important change is distinguished from the change due to measurement error.

The MSWS-12 has been adapted and validated into Persian language (MSWS-12p) [8]. A recent study evaluated the responsiveness of MSWS-12p in PwMS and found a minimally clinically important change (MCIC) of 7.5 based on the summed score of the MSWS-12p items (range score 12–60) [9]. However, the validation study of the MSWS-12p used the total score from “0” to “80”. Based on the data provided in the validation study of the MSWS-12p [8], we calculated the smallest detectable change (SDC) of 8.8 points. This indicates that the MCIC of 7.5 is within the measurement error associated with the MSWS-12p. Further, the authors used the global rating of change score (GRCS) [10] to estimate the MCIC for MSWS-12p. The GRCS is influenced by the patients’ interactions with physiotherapists and may result in an erroneous MCIC, and hence the smallest detectable change (SDC) is recommended for receiver operator characteristic (ROC) analysis [11]. We aimed to reevaluate the responsiveness of MSWS-12p in PwMS according to the scoring system used in the previous validation study of the MSWS-12p and use the SDC criterion for ROC analysis to determine the MCIC.

## Materials and methods

### Design and participants

PwMS who were referred to Physiotherapy Clinic of Iran MS Society were included in the study. This prospective, pretest-posttest study was conducted from March 2019 to March 2020 in Tehran, Iran.

Inclusion criteria were: (1) ability to walk with or without walking aids (Expanded Disability Status Scale, EDSS score 4.5–7.5); (2) ability to read and write in Persian; (3) ability to follow the commands; and (4) giving consent to participate in the study. Exclusion criteria were: (1) presence of other neurological disorders; (2) not completing the physiotherapy program; and (3) not completing the all questionnaire items.

### Procedure

Patients willing to participate in the study were evaluated for eligibility. Demographic and basic characteristics of patients including age, gender, body mass index (BMI), and years since MS diagnosis were recorded. The patients were then asked to complete the MSWS-12p questionnaire. Thereafter, patients were tested using the T25-FW before initiating physiotherapy. Physiotherapy in the form of exercise therapy was presented to the patients, three days per week, and 45 min for each session. The physiotherapy treatment was individualized for each patient and consisted of muscle stretching, general conditioning exercises, walking and balance exercises. At the end of the 10th session of physiotherapy, the participants completed the MSWS-12p questionnaire and were re-tested for the T25-FW.

The MSWS-12p is a patient-reported outcome measure that includes 12 items, each item is scored from “1” (not at all) to “5” (extremely affected) with the summed score ranging from 12 to 60. The total score from 0 to 80 was calculated [patient’s score – 12 (minimum score possible)/60 (the maximum score) ×100] [8]. The higher scores on MSWS-12p indicated greater walking disability.

.

The T25-FW is a measure of walking speed [12, 13]. The patient was asked to walk 25 feet (7.62 m) as quickly as possible from a clearly marked start line to a clearly marked finish line. A stopwatch was used to measure the time in seconds. The use of an assistive device, such as a cane, was allowed; the same walking device was used at the follow-up session after treatment. The average of two completed trials was used as the patient’s score. A higher time on the T25-FW indicated greater walking disability.

## Statistical analysis

The mean and standard deviation (SD) were calculated for quantitative variables. Kolmogorov-Smirnov (KS) test was used to examine the normality of the data. Paired T-tests were used to examine the change before and after physiotherapy. The standardized effect size (SES) was used to calculate the effect size (M_pre_ – M_post_/SD_pre_). The standardized response mean (SRM) was calculated by dividing the mean change scores by the SD of change scores. Effect sizes of 0.2, 0.5, and 0.8 were interpreted as small, medium, and large, respectively [14]. Pearson’s correlation coefficient was calculated to determine the association between the MSWS-12p and the T25-FW. Correlation threshold of > 0.30 was considered as acceptable [15]. Eta Coefficient test was used to examine the strength of association between the MSWS-12p total score change and the SDC based categorical variable of “Improved” and “Unimproved”. The correlation coefficients and Eta coefficient test statistic were interpreted Excellent (0.81-1.00); Very good (0.61–0.80); Good (0.41–0.60); Fair (0.21–0.40); and Poor (0.00-0.20) [16].

The ROC was used to evaluate the responsiveness of MSWS-12p. Sensitivity and specificity were defined as the ability of the MSWS-12p to discriminate the “Improved” (scores > 8.8) from “Unimproved” (< 8.8) patients according to the SDC cut-off of 8.8 points for MSWS-12p; Independent-Samples T-Tests was used to examine the difference between two groups on the MSWS-12p and T25-FW mean change scores [11]. The area under the curve AUC was calculated and represents the probability that the MSWS-12p correctly discriminates the patients as improved or unimproved. The AUC of at least 0.7 was considered as sufficient [17]. The SPSS version 18 (SPSS Inc., Chicago, IL, USA) was used for analyzing the data. P-value ≤ 0.05 was considered statistically significant.

## Results

Thirty patients with a mean age of 43.1 years (SD = 9.1, range = 22–59) participated in the study, of whom 16 were female. The mean duration of MS disease was 13.75 years (SD = 6.31). The mean BMI of participants was 23.97 (SD = 4.6).

The mean and SD for MSWS-12p and T25-FW with effects sizes are shown in Table 1. There were significant improvements in the MSWS-12 scores after physiotherapy (p = 0.008); however the change scores for T25-FW were not significant (p = 0.26). The results on MSWS-12p showed that 11 patients improved (mean change 40.30 ± 20.92) and 19 patients did not (-2.54 ± 8.06); the difference between the two groups was statistically significant (t=-6.52, df = 11.74, p < 0.001). The mean change on the T25-FW was not significant between the two groups improved and unimproved (t = 0.81, df = 28, p = 0.42). The correlation between MSWS-12p and T25-FW mean change was not significant (r = -0.14, p = 0.45). The Eta coefficient test showed an excellent association between MSWS-12p total score change and categorical variable of improvement (Eta = 0.84).

**Table 1:** Mean ± standard deviation (SD), Minimum-Maximum, for the Persian Multiple Sclerosis Walking Scale-12 (MSWS-12p) and Timed 25-Foot Walk (T25-FW) scores before and after physiotherapy (N = 30)

Measures	Before	After	Change	Paired *t*-test, *p* value	SES	SRM
**MSWS-12p**	35.33 ± 23.27 0.0-78.33	22.17 ± 17.69 0.0–80.0	13.17 ± 25.14 -21.67-75.0	2.87, 0.008	0.57	0.52
**T25-FW(sec)**	16.10 ± 14.25 5.18-69.0	14.66 ± 11.12 5.33–43.96	1.41 ± 5.51 -6.87-25.04	1.40, 0.17	0.10	0.26

SES, standardized effect size; SRM, standardized response mean

**Fig. 1:**
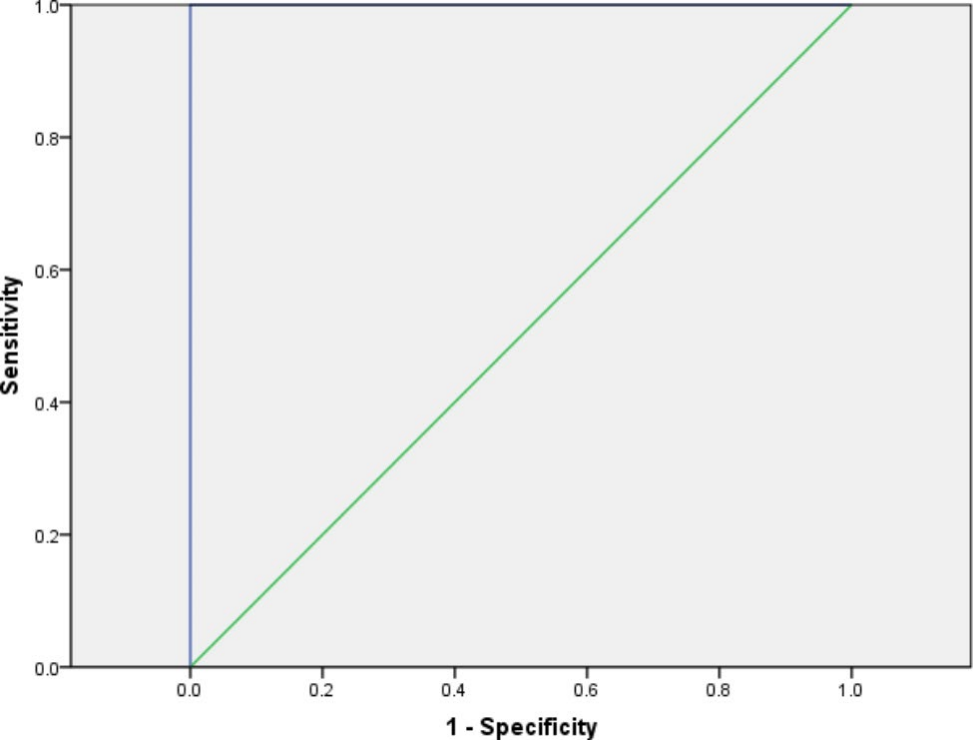
Receiver operating characteristics curve for the Persian Multiple Sclerosis Walking Scale-12 (MSWS-12).

ROC analysis of the MSWS-12p showed the AUC = 1.0. The best MCIC for MSWS-12p was 10.0 points (both sensitivity and specificity 100%) to distinguish PwMS who had an improvement in walking from those who had not (Fig. 1).

## Discussion

The current study aimed to reevaluate the responsiveness of the MSWS-12p and determine the MSWS-12p MCIC to observe whether the prior findings recur. In this study, both distribution-and anchor-based methods were applied [18, 19]. The beneficial effects of physiotherapy on walking were demonstrated in significant changes of MSWS-12p scores similar to previous reports [20, 21] and recent studies on the MSWS-12 responsiveness [9, 22–24]. The improvements after physiotherapy in PwMS support the validity and responsiveness of MSWS-12p within the clinical context. The effect size measures of SES and SRM were not evaluated in previous works [9, 22, 23]. The T25-FW did not improve significantly after physiotherapy [6, 25–28]. The MSWS-12 may be a more appropriate measure than the T25-FW in detecting improvements after physiotherapy [22].

There was a non-significant correlation between the MSWS-12p and the T25-FW consistent with a previous investigation [29]. This indicates that the perceived improvement according to the MSWS-12p is not consistent with the T25-FW measure. However this is not in line with previous reports that found a moderate correlation between the two measures in community PwMS [24, 30]. Poor correlation between the MSWS-12p and the T25-FW suggests they measure different aspects of walking from different perspectives [29].

The correlation between the MSWS-12p and the SDC dichotomized category was excellent and supports its use in identifying patients who are truly improved from those who are unimproved. The SDC implies that the effect of intervention in PwMS using the MSWS-12p as the outcome measure has to be greater than the 8.8 points [8]. Significant differences between the two groups, Improved vs. Unimproved, on the MSWS-12p change scores implies the sensitivity of the MSWS-12p in identifying patients who truly improved after physiotherapy. It follows that the SDC score may be an objective and suitable approach to identify true change in the clinical status of patients after an intervention [11].

In this study, the SDC based analysis of ROC produced a perfect AUC. As well, the MCIC value of 10 points based on SDC criterion for MSWS-12p found in this study is beyond the measurement error. In line with our finding an MCIC of 10.4 was reported previously [22]. However, some reported MCIC of 8.8 [23] and 8 points in PwMS [31]. Recently, the MCIC of 7.5 points reported for the MSWS-12p [9]. A study determined the appropriate and responsive measures based on the AUC > 0.5 and MCIC > SDC as criteria [23]. It follows that the MCIC value must be calculated considering the SDC value of an instrument being evaluated [32, 33].

## Conclusions

The MSWS-12p was found to be responsive when applied in PwMS. Based on the SDC value, the MCIC was calculated as 10 points and is beyond the measurement error of 8.8 points. A change of at least 10 points on the MSWS-12p must occur to be considered as an important clinically meaningful change. Further investigations should confirm our approach and MCIC value.

## Limitations

First, the sample size was small. At least 50 PwMS is necessary according to COSMIN guideline [34, 35]. We had planned to include at least 50 patients but due to the COVID-19 pandemic and limited time for data gathering, we were not able to continue our study. Second, responsiveness was not determined according to the disability level of PwMS. Third, the study included a physiotherapy program in an outpatient setting, meaning that the results are relevant to an outpatient setting.

## Data Availability

The datasets used and/or analyzed during the current study are available from the corresponding author on reasonable request.

## Abbreviations

MS: Multiple sclerosis
MSWS-12: Multiple Sclerosis Walking Scale-12
PwMS: Patients with MS
MCIC: minimally clinically important change
T25-FW: Timed 25-Foot Walk test
AUC: under receiver operating characteristics curve
SDC: smallest detectable change
GRCS: global rating of change score
ROC: receiver operator characteristic
EDSS: Expanded Disability Status Scale
BMI: body mass index
SD: standard deviation
KS: Kolmogorov-Smirnov
SES: standardized effect size
SRM: standardized response mean
COSMIN: COnsensus-based Standards for the selection of health status Measurement Instruments
COVID-19: coronavirus disease of 2019.
